# Novel histological repertoire of crypt-associated anomalies in inflamed colon mucosa

**DOI:** 10.1136/jclinpath-2022-208152

**Published:** 2022-03-10

**Authors:** Carlos A Rubio, Michael Vieth, Corinna Lang-Schwarz

**Affiliations:** 1 Department of Pathology, Karolinska Institute and University Hospital, Stockholm, Sweden; 2 Institute of Pathology, Friedrich-Alexander University Erlangen-Nuremberg, Klinikum Bayreuth, Bayreuth, Germany

**Keywords:** colitis, inflammation, inflammatory bowel diseases, intestine, large

## Abstract

**Aims:**

Studying crypt branching in ulcerative colitis (UC) and in infectious colitis (IC), we detected previously unreported crypt-associated anomalies (CAAs). The objective was to describe, illustrate and assess the frequency of CAAs in inflamed colon mucosa in patients with UC and IC.

**Methods:**

Sections from 100 consecutive biopsies with UC, in 50 with IC and in 27 with UC in remission (UCR) were reviewed. The following CAAs were identified: crypt eosinophilia, intracryptal epithelial hyperplasia, intracryptal epithelial budding, intracryptal supernumerary crypts, intracryptal epithelial bridges, crypt rings in rows and off-centre epithelial budding.

**Results:**

The frequency of crypts with extensive crypt eosinophilia and with intracryptal epithelial budding was significantly higher in UC than in IC and UCR (p<0.05); the frequency in the remaining histological parameters was similar in UC, IC and UCR.

**Conclusions:**

CAAs were found interspersed with branching crypts. CAAs persisted in long-lasting UC mucosal inflammation, but declined when the inflammation waned. Since similar anomalies are not present in normal colon mucosa, the results suggest that CAAs had been boosted by the ongoing mucosal inflammation. The development of these previously unreported CAAs in the colon mucosa with inflammation might embody pathological aberrations of cryptogenesis.

## Introduction

The sagittal profile of the normal colon mucosa at low magnification discloses a wavy silhouette due to small mucosal protrusions called *anthemia*, a Greek term used to define alternating decorative motifs.^1^ An *anthemion* is built of parallel close mucosal invaginations called crypts. In well-oriented sections, the crypts are arranged perpendicular to the surface epithelium and to the *muscularis mucosae*, as ‘test tubes’ in a rack.^2^ In the middle of the *anthemion*, the crypts are the tallest, but decrease in height, sideways. This explains the slight variations in the height of the crypts found in well-oriented tissue sections.^1^ The *anthemia* are limited by furrows called innominate grooves.^3^

In early infancy, the building of new crypts is achieved by symmetric branching or fission.^4^ Building of new crypts begins at their base with a crevice; the cleft progresses upwards until two identical crypts are eventually formed.^5^ During late infancy and adulthood, crypt replication wanes; crypts divide only once or twice, with average crypt cycle length of 36 years.^4^ Thus, crypt branching in the colon of adults is a rare finding.^6–8^ In contrast, the colonic mucosa in patients with inflammatory bowel disease (IBD), including ulcerative colitis (UC) and Crohn colitis, displays increased number of branching crypts.^9^ Several authors claim that branching can easily be detected in crypts cut in the vertical plane^6 10 11^ (ie, in well-oriented, upright crypts), but not in those cut in the horizontal plane (ie, in cross-cut crypts) as they ‘often produced acinar profiles’.^11 12^ [Bibr R12] However, daily praxis shows that sections from endoscopic biopsies are fortuitously cut at the laboratory in a horizontal plane, a procedure often yielding cross-cut ring-shaped crypts. More recently, Rubio *et al* 13–16 reported the occurrence of increased numbers of branching crypts in endoscopic biopsies from patients with IBD. The presence of epithelial crests in ring-shaped cross-cut crypts identified the beginning of crypt branching.^16^ Their results showed that the number of crypts in asymmetric branching surpassed that of crypts in symmetric branching.^14^

Recently, while reviewing sections from patients with UC, a series of previously unreported crypt-associated histological anomalies were detected; they were regarded as histological artefacts. Notwithstanding, the review of endoscopic biopsies from patients with infectious colitis (IC) revealed similar artefacts. Against this background, the following questions arose:

- Could those anomalies merely mirror crypt alterations conveyed by the mucosal inflammation per se?
- Could one or more of those anomalies be pathognomonic of UC?

The aim of the present investigation was to explore these questions by analysing a cohort of patients with UC, IC and UC in remission (UCR).

## Materials and methods

Biopsies from 100 consecutive patients with UC under surveillance and from 79 controls (50 with IC and 27 with UCR) were analysed. Following the local endoscopic scheme, a minimum of two endoscopic biopsies were taken from various colon segment and rectum. Biopsies were cut in 4 µm thick sections, stained with H&E, scanned with a Hamamatsu NanoZoomer S360 digital scanner and made available online to authors.

### Novel histological parameters

The following histological parameters were found in the crypts in patients with colon inflammation: (1) crypt eosinophilia, (2) intraepithelial cell hyperplasia, (3) intracryptal epithelial budding, (4) intracryptal supernumerary crypts, (5) intraluminal epithelial bridges, (6) crypt rings in rows and (7) off-centre epithelial budding.

### Definitions

#### Crypt eosinophilia

**Figure 1:**
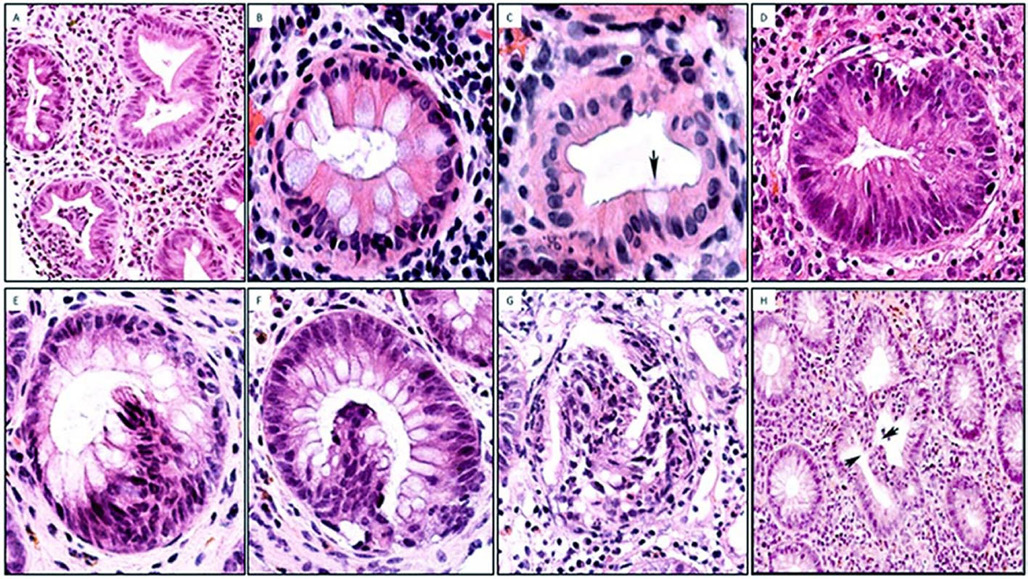
Crypt eosinophilia, intraepithelial cell hyperplasia and intracryptal budding found in inflamed colon mucosa. (A) Colon crypts with eosinophilia. Note the occasional goblet cells (H&E, original ×4). (B) Detail of crypt eosinophilia showing eosinophilia in columnar cells (H&E, original ×20). (C) Another crypt with eosinophilic cells with one goblet cell (arrow) (H&E, original ×20). (D) Colon crypt with intense eosinophilia (H&E, original ×20). (E) Crypt with intraepithelial cell hyperplasia. Note the focal nuclear stratification (H&E, original ×20). (F) Crypt with intracryptal budding protruding into the crypt lumen (H&E, original ×20). (G) Another view from a crypt with intracryptal budding protruding into the crypt lumen (H&E, original ×10). (H) Crypt with two ‘face-to-face’ intracryptal buddings protruding into the crypt lumen. Note the small incomplete gap between the two arrows (H&E, original ×4).

Under normal conditions, colon crypts are mainly lined with non-eosinophilic, mucus-producing goblet cells, which compress sideways columnar absorptive cells. Goblet cells decrease towards the upper portion of the crypts, and near the luminal portion of the crypts cells with eosinophilic cytoplasm are usually seen. Scrutiny of crypt cells revealed that crypt cells with intense eosinophilic cytoplasm were the columnar absorptive cells (figure 1A–D). Crypt eosinophilia was classified by semiquantitative estimates into (1) focal crypt eosinophilia (ie, haphazardly distributed eosinophilic crypts) and (2) extensive crypt eosinophilia (ie, crypt eosinophilia found in most crypts).

#### Intraepithelial cell hyperplasia

These are intraepithelial hubs showing hyperplastic epithelium, easily recognised by focal nuclear stratification (figure 1E).

#### Intracryptal epithelial budding

**Figure 2:**
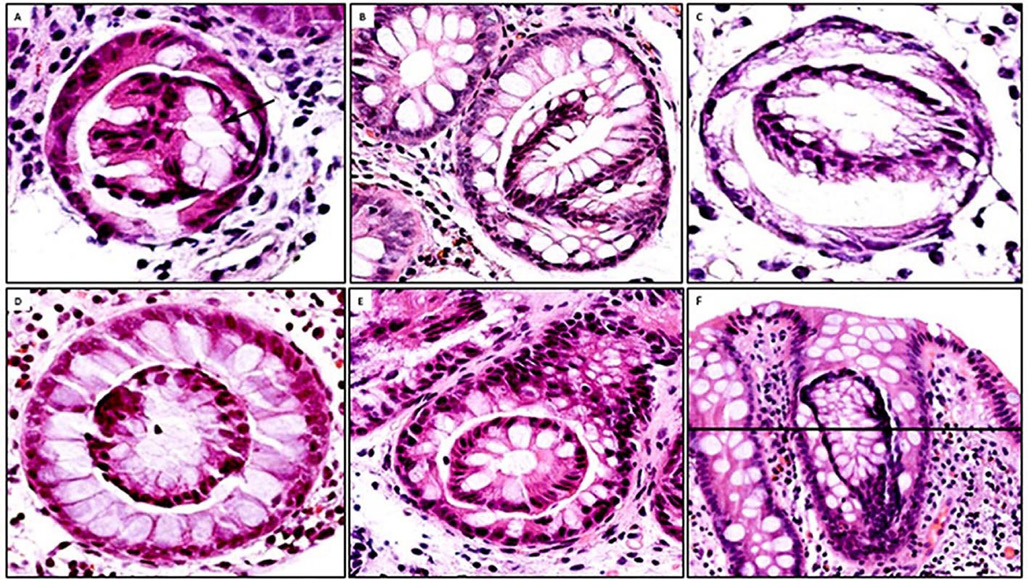
Putative pathway from intracryptal budding with a minor central lumen to ‘crypt-in-crypt’ found in inflamed colon mucosa. (A) Intracryptal budding with a minor central lumen (arrow) (H&E, original ×20). (B, C) Intracryptal budding connected with the ‘mother’ crypt; note the large central lumen (H&E, original ×20). (D) ‘Crypt-in-crypt’ without apparent connection with the ‘mother’ crypt (H&E, original ×20). (E) Another view of ‘crypt-in-crypt’ without apparent connection with the ‘mother’ crypt (H&E, original ×10). (F) Vertical crypt with a transversal line; a histological section cut at that level unveils the ‘crypt-in-crypt’ mystery (H&E, original ×10).

These are epithelial growths bulging into the crypt lumen developing from a ‘mother’ crypt (figure 1F–H). Some intracryptal epithelial budding revealed a minor central lumen (figure 2A); other intracryptal epithelial budding develops into more distinct crypts attached to the ‘mother’ crypt (figure 2B,C).

#### Intracryptal supernumerary crypts

**Figure 3:**
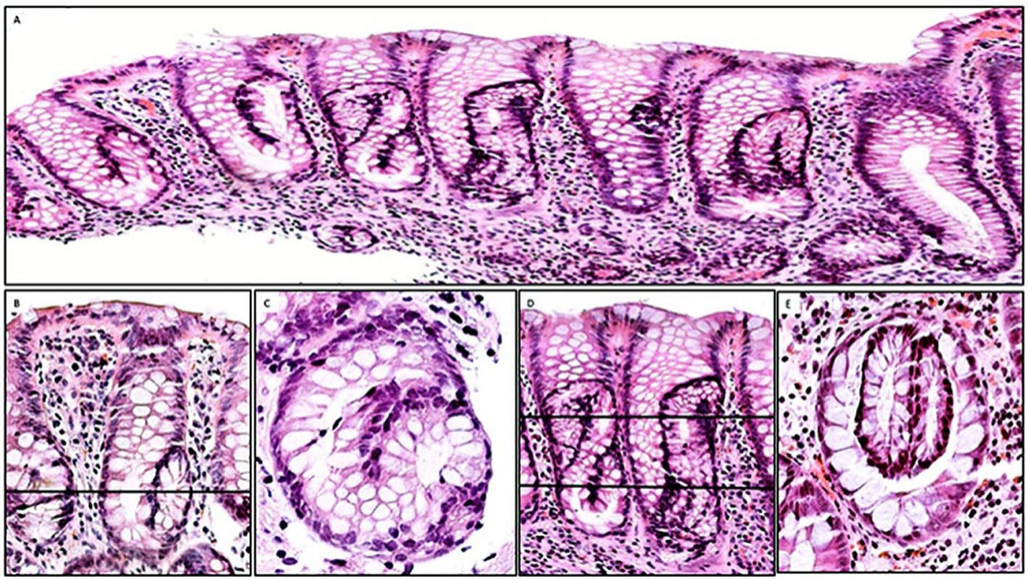
Intracryptal supernumerary crypts found in inflamed colon mucosa. (A) Low-power view colon biopsy showing vertical crypts with bizarre crypts growing inside the ‘mother’ crypt. Note that the internal crypts assume U-turn or pretzel/8-like configurations (H&E, original ×4). (B) A closer view of vertical crypts with intracryptal supernumerary crypts unveiling U-bent configuration and a transversal line; a histological section cut at that level would reveal one cross-cut crypt with a pseudo-epithelial bud (C). (D) Vertical crypts with bizarre pretzel/8-like profiles and a transversal line; a histological section cut at that level would reveal cross-cut ‘two-crypts-in-crypts’ (E) (B, D: H&E, original ×10; C, D: H&E, original ×20).

In cross-cut sections, intracryptal supernumerary crypts were epitomised by a luminal crypt ring without apparent connection with the ‘mother’ crypt (figure 2D,E). Figure 2F shows a crypt with a transversal line; a histological section cut at that level unveils the ‘crypt-in-crypts’ mystery, such as in figure 2D,E. Growing intracryptal supernumerary crypts adopted bizarre pretzel or 8-like profiles (figure 3A). Figure 3B shows a bent crypt with U-turn configurations and a transversal line; a histological section cut at that level would reveal one cross-cut crypt with a pseudo-epithelial bud (figure 3C). Figure 3D shows vertical crypts with bizarre profiles and a transversal line; a histological section cut at that level would reveal cross-cut ‘two-crypts-in-crypts’ (figure 3E).

#### Intraluminal epithelial bridges

**Figure 4:**
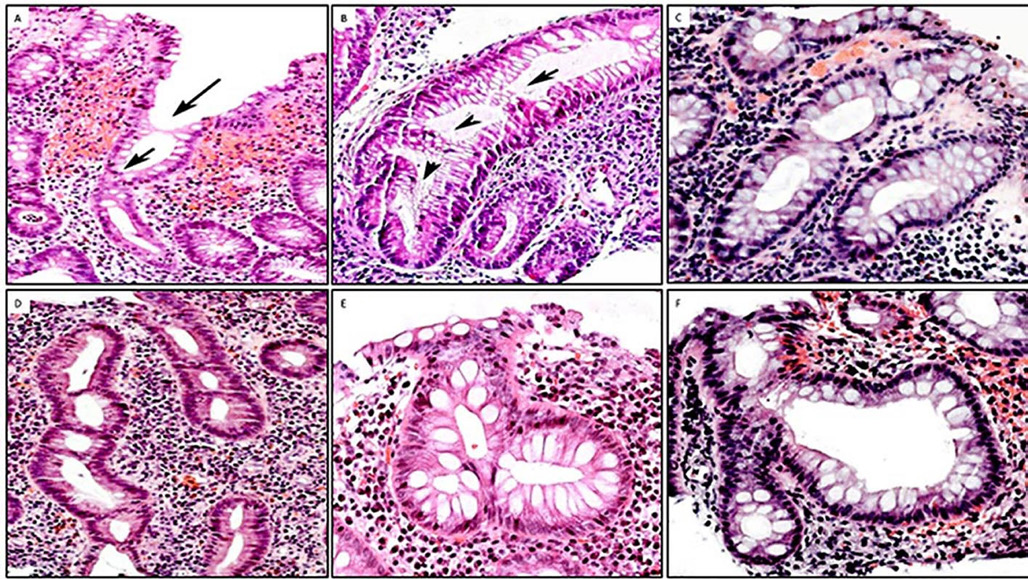
Intraluminal epithelial bridges, crypt rings in rows and off-centre epithelial budding found in inflamed colon mucosa. (A) Two bands of epithelium (arrows) across the crypt lumen, dividing the lumen into ≥2 compartments (H&E, original ×10). (B) Closer view of crypts with intraluminal epithelial bridges (H&E, original ×20). (C) Crypts in rows in the upper portion of the colon mucosa (H&E, original ×10). (D) Another view of crypts in rows or files in the upper portion of the colon mucosa (H&E, original ×10). (E, F) Close view of two crypts with off-centre epithelial budding (H&E, original ×20).

These are one or more bands of epithelium found across the crypt lumen, dividing the lumen into ≥2 compartments (figure 4A,B).

#### Crypt rings in rows

These are crypts with several intraluminal epithelial bands, building a chain of crypt rings in file (figure 4C,D).

#### Off-centre epithelial budding

These are buds merging from the lateral aspect of a crypt, bulging into the *lamina propria.* Off-centre epithelial budding was mainly found in vertically oriented sections (figure 4E,F).

#### Degree of mucosal inflammation

Mucosal inflammation was classified by semiquantitative estimates into (1) slight-moderate mucosal inflammation and (2) severe mucosal inflammation.

### Statistical analysis

Non-parametric Mann-Whitney U two-tailed test was applied to compare differences between two independent groups. Statistical significance was defined as p<0.05.

## Results

The results are condensed in table 1.

**Table 1:** Frequency of crypt-associated anomalies found in H&E-stained sections of 100 cases with ulcerative colitis, 50 cases with infectious colitis and 29 cases with ulcerative colitis in remission

Crypt-associated anomalies	Ulcerative colitis (n=100)	Infectious colitis (n=50)	Ulcerative colitis in remission (n=29)
Extensive crypt eosinophilia	58	10	0
Intracryptal epithelial hyperplasia	23	7	1
Mean	0.23	0.14	0.03
Range	0–3	0–2	
Intracryptal epithelial budding	191	28	5
Mean	1.9	0.56	0.17
Range	0–11	0–4	0–1
Intracryptal supernumerary crypts	41	19	2
Mean	0.4	0.38	0.07
Range	0–13	0–7	
Intraluminal epithelial bridges	5	2	0
Mean	0.05	0.04	
Range	0–1		
Crypt rings in rows	12	3	0
Mean	0.12	0.06	
Range	0–1	0–1	
Off-centre epithelial budding	17	3	0
Mean	0.17	0.06	
Range	0–6	0–1	
Severe mucosal inflammation (%)	52 (52)	13 (26)	0

### Crypt eosinophilia

From table 1 it may be deduced that of the 58 cases with extensive crypt eosinophilia, 58% were found in UC, 20% in IC and none in UCR. The differences between extensive crypt eosinophilia in UC, IC and UCR were significant at p<0.05 (p=0.00016 and p=0.00001, respectively).

### Intracryptal epithelial hyperplasia

Out of a total of 31 crypts with intracryptal epithelial hyperplasia in table 1, 74% had UC, 23% IC and 3% UCR. The differences between UC on the one hand and IC and UCR on the other were *not* significant at p<0.05 (p=0.53526 and p=0.17384, respectively).

### Intracryptal epithelial budding

Of a total of 224 crypts with intracryptal epithelial budding recorded, 85% were found in UC, 13% in IC and 2% in UCR. The differences between UC on the one hand and IC and UCR on the other were significant at p<0.05 (p<0.00008 and p<0.00001, respectively). Hence, majority of the crypts with epithelial cores were found in UC.

### Intracryptal supernumerary crypts

A total of 62 crypts with intracryptal supernumerary crypts were found; 66% were found in UC, 31% in IC and 3% in UCR. The differences between UC and both IC and UCR were *not* significant at p<0.05 (p<0.92828 and p<0.6672, respectively).

### Intraluminal epithelial bridges

Majority (71%) of the seven intraluminal epithelial bridges recorded were found in UC and the remaining 29% in IC. No intraluminal epithelial bridge was found in UCR.

### Crypt rings in rows

Out of the 15 crypt rings in rows found, majority (80%) occurred in UC and the remaining 20% in IC. No crypt rings in rows were found in UCR. The difference between UC and IC was *not* significant at p<0.05 (p=0.31732).

### Off-centre epithelial budding

Out of a total of 20 off-centre epithelial budding detected, majority (85%) occurred in UC and the remaining 15% in IC. No off-centre epithelial budding was found in UCR. The difference between UC on the one hand and IC and UCR was *not* significant at p<0.05 (p=0.27572 and p=0.16452, respectively).

### Mucosal inflammation

Table 1 also shows that severe mucosal inflammation was found in 52% of cases with UC, in 26% with IC but in none of those with UCR. The difference between severe mucosal inflammation in UC, IC and UCR was significant at p<0.05 (p=0.01174 and p=0.00001, respectively).

## Discussion

Most reports in the literature concur that the histological parameters found in colon UC include, besides inflammation, ulcer/erosion, goblet cell depletion, crypt distortion/atrophy, villiform changes on the surface epithelium, basal plasmacytosis, basal lymphoid aggregates and Paneth cell metaplasia.^17 18^ In this survey, a series of previously *un*reported histological parameters evolving in the colon mucosa with inflammation are presented, namely crypt eosinophilia, intraepithelial cell hyperplasia, intracryptal epithelial budding, intracryptal supernumerary crypts, intraluminal epithelial bridges, crypt rings in rows and off-centre epithelial budding.

Extensive crypt eosinophilia was found significantly higher in UC than in IC. Eosinophilia is conveyed by eosin, an anionic-acidic dye that tints pink or red all acidophilic cells.^19 20^ If epithelial eosinophilia is the result of goblet cell depletion, then epithelial eosinophilia should have been conveyed by the remaining columnar absorptive cells. On the other hand, we have not seen crypts with goblet cell depletion having smaller luminal diameter than crypts rich in goblet cells. Against this background, the following options should be considered to possibly explain epithelial eosinophilia in the crypts:

- That columnar cells proliferate to maintain the luminal diameter of the crypts. This option was discarded since columnar cells are highly differentiated cells and unable to divide.
- That columnar absorptive cells together with the cytoplasmic skeleton of goblet cells without mucus might have contributed to the epithelial eosinophilia of the crypts.

The latter might be a possible alternative.

**Figure 5:**
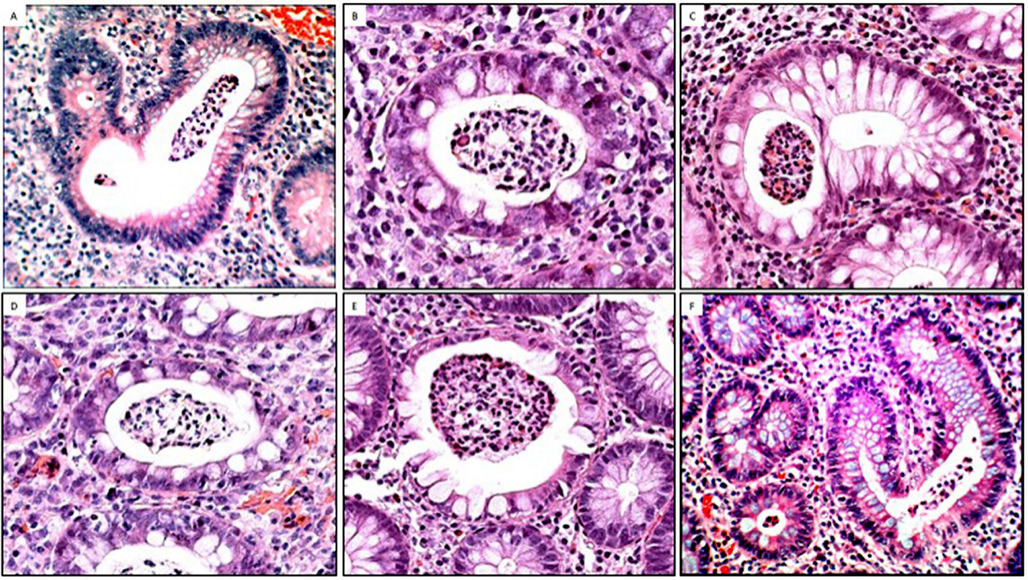
Presence of goblet cells in colon crypts with inflammatory abscesses. (A–F) Examples of crypts with abscesses exhibiting various proportions of goblet cells. Despite active inflammation, crypts display various amounts of goblet cells (A, B, C, D, E: H&E, original ×20; F: H&E, original ×10).

To assess whether acute inflammation influenced the goblet cell population, we classified biopsy cases with UC into those with or without crypt abscesses. The results showed that out of the 100 biopsy/cases with UC, 20 exhibited crypt abscesses; of these 45% (9 of 20) showed total goblet cell depletion and the remaining 55% (11 of 20) showed various proportions of goblet cells (figure 5). Thus, total goblet cell depletion is not the rule in colon crypts in patients with UC with active inflammation.^19 20^

Unlike histologically normal crypts, some crypts with mucosal inflammation (UC and IC) or without inflammation (UCR) displayed intracryptal epithelial hubs showing focal cell stratification. These hubs of cell hyperplasia might be the first step leading to intracryptal budding protruding into the crypt lumen.

Intracryptal buddings were found significantly more frequently in UC than in IC and UCR. Importantly, the central microlumen found in some intracryptal epithelial buds suggests that the growth of crypts with microlumen could progress to intracryptal supernumerary crypts. Thus, focal cell stratification and epithelial budding via crypt-in-crypts may materialise the sequence of histobiological events taking place in colon mucosa with inflammation. Why some crypts develop intracryptal budding while others grow outer cryptal budding (ie, off-centre budding) remains elusive.

Another crypt-associated anomaly found was the building of one or more transluminal epithelial bridges, which transformed the original crypt lumen into several smaller lumina. This histological assemblage, mostly found in the upper portion of the mucosa, was called crypt rings in rows.

It might be argued that crypt-associated anomalies are histological ‘artefacts’ of preparation. However, there was no indication for that claim, since neither ‘artefacts’ nor similar crypt-associated anomalies have been recorded in normal colon mucosa in humans^2 20–22^ or in laboratory animals.^23 24^

Of the crypt-associated anomalies reported here, only crypt eosinophilia and intracryptal epithelial budding were significantly higher in UC than in IC or UCR; in the remaining histological parameters, no difference was found between UC and IC. These similarities between UC and IC strongly suggest that crypt-associated anomalies are triggered by mucosal inflammation as such. But importantly, IC lasts only a few weeks and those anomalies disappear almost entirely when mucosal inflammation wanes. On the other hand, crypt-associated anomalies persist in long-lasting, protracted inflammation.

In attempts to possibly explain the histological conundrum evolving in crypts of patients with colonic inflammation, the following options appear pertinent:

- That crypt-associated anomalies highlight the pathological cryptogenesis that evolves in the colon mucosa with inflammation. This possibility seems to be validated by the finding of crypt-associated anomalies interspersed with crypts in asymmetric branching.^16^
- That crypt-associated anomalies never reach the stadium of asymmetric branching as they are digested by mast cells and/or gut microbiome proteases.^25 26^

In conclusion, this survey describes a series of previously unreported anomalies evolving in crypts from the colon mucosa with inflammation. These easy-to-identify anomalies were found interspersed with branching crypts. Since similar anomalies are not present in the colon mucosa of individuals without inflammation,^2^ it is rational to assume that the development of crypt-associated anomalies in the colon mucosa with inflammation embodies pathological aberrations of cryptogenesis.

Further research is necessary before the true significance of these probably scheduled pathological aberrations of cryptogenesis affecting the colon mucosa with inflammation can be fully understood.

**Take home messages**

- According to the literature, inflammation of the colon mucosa leads to architectural distortions of the crypts.
- This study describes previously unreported crypt-associated anomalies found in the colon mucosa with inflammation.
- Crypt-associated anomalies in colon inflammation might embody pathological aberrations of cryptogenesis.
- The study might open new vistas in the process of crypt branching in inflamed colon.

## Data Availability

Data are available upon reasonable request.
